# Clinical statistical analysis plan for the ACCURE trial: the effect of appendectomy on the clinical course of ulcerative colitis, a randomised international multicentre trial

**DOI:** 10.1186/s13063-024-08037-5

**Published:** 2024-03-26

**Authors:** Eva Visser, Lianne Heuthorst, Shri Pathmakanthan, Willem A. Bemelman, Geert R. D’Haens, Kelly Handley, Apostolos Fakis, Thomas D. Pinkney, Christianne J. Buskens, Marcel G. W. Dijkgraaf

**Affiliations:** 1 Department of Surgery, Amsterdam University Medical Centre, Amsterdam, The Netherlands; 2 Department of Gastroenterology, Queen Elizabeth University Hospital Birmingham, Birmingham, UK; 3 Department of Gastroenterology and Hepatology, Amsterdam University Medical Centre, Amsterdam, the Netherlands; 4 University of Birmingham Clinical Trials Unit, Birmingham, UK; 5 Derby Clinical Trials Support Unit, University Hospitals of Derby and Burton NHS Foundation Trust, Derby, UK; 6 Academic Department of Surgery, University of Birmingham, Birmingham, UK; 7 Department of Epidemiology and Data Science, University Medical Centre, Amsterdam, the Netherlands; 8 Methodology, Amsterdam Public Health, Amsterdam, the Netherlands

**Keywords:** Statistical analysis plan, Inflammatory bowel disease, Ulcerative colitis, Appendectomy, Disease recurrence

## Abstract

**Background:**

The primary treatment of ulcerative colitis (UC) is medical therapy using a standard step-up approach. An appendectomy might modulate the clinical course of UC, decreasing the incidence of relapses and reducing need for medication. The objective of the ACCURE trial is to assess the efficacy of laparoscopic appendectomy in addition to standard medical treatment in maintaining remission in UC patients. This article presents the statistical analysis plan to evaluate the outcomes of the ACCURE trial.

**Design and methods:**

The ACCURE trial was designed as a multicentre, randomised controlled trial. UC patients with a new diagnosis or a disease relapse within the past 12 months, treated with 5-ASA, corticosteroids, or immunomodulators until complete clinical and endoscopic remission (defined as total Mayo score < 3 with endoscopic subscore of 0 or 1), were counselled for inclusion. Also, patients previously treated with biologicals who had a washout period of at least 3 months were considered for inclusion. Patients were randomised (1:1) to laparoscopic appendectomy plus maintenance treatment or a control group (maintenance therapy only). The primary outcome is the 1-year UC relapse rate (defined as a total Mayo-score ≥ 5 with endoscopic subscore of 2 or 3, or clinically as an exacerbation of symptoms and rectal bleeding or FCP > 150 or intensified medical therapy other than 5-ASA therapy). Secondary outcomes include number of relapses per patient, time to first relapse, disease activity, number of colectomies, medication usage, and health-related quality of life.

**Discussion:**

The ACCURE trial will provide comprehensive evidence whether adding an appendectomy to maintenance treatment is superior to maintenance treatment only in maintaining remission in UC patients.

**Trial registration:**

Dutch Trial Register (NTR) NTR2883. Registered May 3, 2011. ISRCTN, ISRCTN60945764. Registered August 12, 2019.

**Supplementary Information:**

The online version contains supplementary material available at 10.1186/s13063-024-08037-5.

## Introduction

### Background and rationale

Ulcerative colitis (UC) is a chronic inflammatory bowel disease (IBD) affecting the mucosa of the colon and rectum, with an annual incidence of 6–8 new cases per 100,000 [1]. The primary treatment is medical therapy consisting of step-up approach starting with 5-aminosalicylic acids (5-ASA), followed by immunomodulators, biologicals, small molecules, and trial medication. Most patients will remain on long-term medication to prevent exacerbations and preserve quality of life. Despite the expanding medical armamentarium and declining emergent UC colectomy rates, the overall incidence of (procto)colectomy in UC patients has remained unchanged over the years [2]. Nevertheless, up to 20% of the patients require surgery [3, 4].

There is increasing evidence suggesting an immunomodulatory role of the appendix in patients with UC [5, 6]. We hypothesise that an appendectomy has a beneficial effect on the UC disease course: decreasing the number of relapses and reducing the need for (upscaling) medication. The ACCURE trial is a randomised, international, multicentre trial to assess the efficacy of appendectomy to maintain remission in patients with UC [7]. From September 2012 to September 2022, 201 patients were randomised. Analyses will commence in 2023 following completion of 1-year follow-up for the last patient, data cleaning checks, and data lock.

## Objectives

The objective of the ACCURE trial was to determine the efficacy of appendectomy in addition to standard medical treatment to maintain remission in patients with UC and to establish the acceptability of the intervention compared to standard treatment only. The trial protocol was previously published [7]. The present manuscript is the proposed statistical analysis plan (SAP), which follows the JAMA Guidelines for the content of statistical analysis plans in clinical trials (Supplementary Material 1) [8].

## Study methods

### Trial design

The ACCURE trial was an investigator-initiated two-arm, multicentre, randomised controlled superiority trial. UC patients in complete clinical and endoscopic remission (defined as Mayo score < 3 with endoscopic subscore 0 or 1) who were treated for a relapse within the past 12 months (with 5-ASA, corticosteroids, immunomodulators or after a washout period of at least 3 months after treatment with biologicals) were randomised into two groups. The intervention group underwent laparoscopic appendectomy in day care setting plus maintenance medical therapy. The appendix was removed including the cecal base to include the orifice of the appendix using a laparoscopic endostapler. The control group continued maintenance therapy at the discretion of the treating gastroenterologist.

The ACCURE trial included two trial registrations. The ACCURE trial (NL) was registered at the Dutch National Trial Register (NTR2883) on May 3, 2011. Ten centres were involved in the trial in the Netherlands (NL) and Ireland. The ACCURE-UK-2 (ISRCTN60945764) is the UK arm of the ACCURE trial (NL) and was registered on August 12, 2019. The study was conducted in 10 hospitals in the United Kingdom (UK). The ACCURE trial (NL) and ACCURE-UK-2 shared a matched overall study design and form the definitive trial (the ACCURE trial) for the final analysis.

### Randomisation

Eligible patients were randomly assigned (1:1 ratio) by the research team with ALEA randomisation software. Randomisation was stratified by disease localisation (rectum, left-sided colitis, pancolitis). Patients and physicians were not blinded during treatment.

### Sample size

The ACCURE trial (NL) was powered on a clinically relevant reduction in relapse rate from an expected 40% in the control group to 20% in the intervention group [7]. With a 5% two-sided significance level, 82 patients per study arm were needed to achieve 80% power to detect such a difference using chi-square test. Considering 10% patient dropouts, we aimed to include 184 patients in order to analyse 164 patients.

In September 2019, the ACCURE trial was started in the United Kingdom (ACCURE-UK-2) to improve recruitment and increase statistical power. The aim was to include 244 patients intending to analyse 218 patients (109 per study arm) to reach 90% power in demonstrating superiority of appendectomy. However, the study was closed after the inclusion of 201 patients in September 2022 due to prolonged accrual (related to the COVID-19 pandemic).

### Framework

The ACCURE trial was a superiority trial. The hypotheses for the primary analysis were as follows:

- Null hypothesis: there is no difference in the 1-year cumulative relapse rate between laparoscopic appendectomy plus maintenance therapy versus maintenance therapy only.
- Alternative hypothesis: there is a difference in the 1-year cumulative relapse rate between laparoscopic appendectomy plus maintenance therapy versus maintenance therapy only.

### Statistical interim analysis and stopping guidance

According to the protocol, no planned interim analysis was scheduled. However, during the inclusion period, a few manuscripts were published suggesting a relation between appendectomy and the development of high-grade dysplasia (HGD) and colorectal cancer (CRC) in UC patients [9]. Therefore, an interim analysis for safety was performed at the discretion of the Data Monitoring and Safety Committee (DSMC) after inclusion of 153 patients in March 2021. In addition to the number of (serious) adverse events in both groups at 1 year, the interim analysis for confirmation of safety also addressed the number of patients with HGD and CRC in both groups during long-term follow-up. For safety regarding neoplasia, the following rules were defined: when the absolute number of patients with HGD/CRC in the intervention group was higher by 1: continuation of the trial; higher by 2: assessment of potential underlying risk factors for HGD/CRC (i.e. onset before adulthood, disease duration > 10 years, concomitant PSC); higher by 3: continuation of the trial was at the discretion of the DSMC. When the absolute number of patients with HGD/CRC was higher in the control group (standard care), assessment of cases could be conducted at the discretion of the DSMC. Conditional on appendectomy being considered safe, the interim analysis was proceeded with a stopping rule for superiority (Haijbittle-Peto boundary *P* < 0.001). In this analysis, no overwhelming efficacy could be demonstrated. The DSMC did not share the outcome results with the research group but communicated that there was no need for early termination of the trial.

### Timing of final analysis

The analyses will be performed when the last patient has reached 1 year follow-up, data entry has been completed, the collected patient data have been monitored, and after this SAP has been accepted for publication.

### Timing of outcome assessments

Outpatient clinic visits or telephone consults were performed at 6 weeks and 3, 6, 9, and 12 months after appendectomy or in the control group after randomisation. During these contacts, the partial Mayo score (pMS), medication use, complications, readmissions, hospital stay, and visits to outpatient clinic were assessed [10]. Health-related quality of life (HRQL) questionnaires (EQ-5D, EORTC-QLQ-C30, and IBDQ) [11–13] were completed at inclusion and every 3 months thereafter during the first year. In the Netherlands, the questionnaires were sent via the MyIBDcoach application or could be completed online. In the UK, hard copies of the questionnaires were completed by the participant on site at the baseline visit or at home and returned by post if an in-person visit was not possible, and at all subsequent time points, the questionnaires were posted out by the central trial team. An endoscopy was performed at the time of suspected relapse or at the end of the 12-month study period (12 months after appendectomy in the intervention group and after randomisation in the control group) to objectively assess mucosal appearance and determine the full Mayo score.

## Statistical principles

### Confidence intervals and *P* values

All statistical tests will be two-sided. *P* values of less than 0.05 will be considered statistically significant. The presented confidence intervals will be 95% and two-sided.

### Adherence and protocol violation

Protocol violation in eligibility was defined as randomisation of a patient who did not qualify for inclusion or who met an exclusion criterion. These patients were excluded from intervention and further follow-up.

**Fig. 1:**
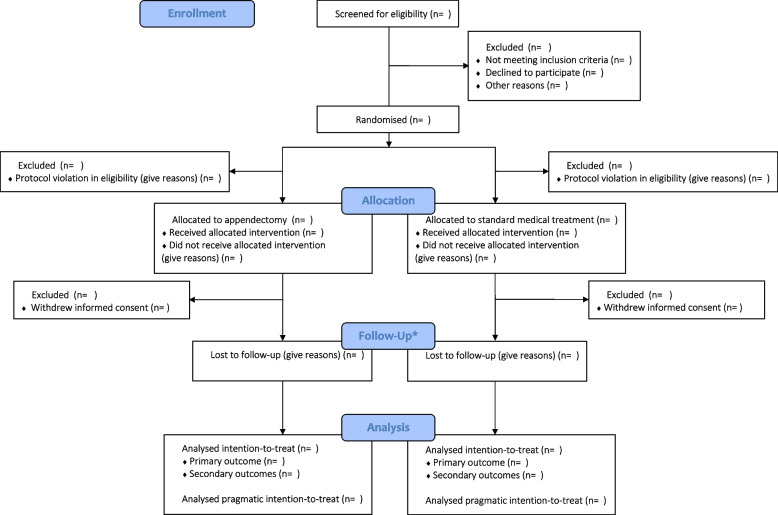
CONSORT flow diagram

Predefined as a major protocol violation with a direct impact on the primary outcome was UC relapse during the waiting period for appendectomy in the intervention group. These patients were not excluded, but the number (and percentage) of patients with a protocol violation will be summarised by group with details of the type of deviation provided and reported in a patient flow diagram according to the Consolidated Standards of Reporting Trials (CONSORT, Fig. 1).

### Analysis populations

All primary analyses (primary and secondary outcomes) will be based on the intention-to-treat (ITT) principle. All randomised patients will be included in the analyses according to their initially assigned study arm at baseline, regardless of whether they actually received the allocated intervention or not. Patients with a protocol violation concerning eligibility will be excluded from analysis. Safety data will be reported by treatment arm, and an as-treated (AT) analysis will be performed. In the AT analysis, patients will be analysed according to the treatment they actually received, rather than the study arm they were initially assigned.

## Trial population

### Screening and eligibility

Patients were screened for eligibility using the inclusion and exclusion criteria according to the most recent version of the study protocol. The number of excluded patients after randomisation and reasons for ineligibility will be reported and illustrated in the CONSORT flow diagram (Fig. 1).

Inclusion criteria:

- Aged ≥ 18 years
- Established diagnosis of UC according to ECCO guideline [14]
- Disease relapse within 12 months prior to randomisation medically treated until remission
- Clinically confirmed remission at time of randomisation, with pMS < 3 and presumptive endoscopic Mayo subscore of 0 or 1, identified by either:
  - Colonoscopy (within 3 months) examining the full length of the colon and rectum
  - Sigmoidoscopy (within 3 months) examining the last part of the colon (sigmoid and rectum) with faecal calprotectin (FCP) < 150 μg/g
  - FCP < 150 μg/g with a personal history of raised FCP levels (> 500 μg/g) during a previous disease flare-up at any stage
- Obtained informed consent

Exclusion criteria:

- Prior appendectomy or major abdominal surgery precluding safe appendectomy
- (Suspicion of) Crohn’s disease
- Disease recently treated with biologicals (within 3 months prior to inclusion)
- pMS ≥ 3 or endoscopic Mayo score > 1
- Medical comorbidity that increases perioperative morbidity

### Recruitment

Informed consent was obtained from the patients according to the ACCURE trial protocol. For both treatment arms, the numbers of patients who were randomised, received the intended treatment, and were analysed for the primary outcome will be presented in the CONSORT flow diagram (Fig. 1).

### Withdrawal/follow-up

For each group, withdrawal and loss to follow-up will be reported and specified with reasons at each time point (Fig. 1). These outcomes will be explored as per other missing responses.

### Baseline patient characteristics

The baseline characteristics of the included patients will be reported per randomisation group and shown in a baseline table (Table 1). Categorical variables will be summarised by numbers and percentages in each category. Continuous variables will be summarised by mean and standard deviation or median and interquartile range, as appropriate. Tests of statistical significance will not be undertaken, nor will confidence intervals be presented [15].

**Table 1:** Baseline characteristics of the patients included in the trial (intention to treat)

*Characteristic*	*Appendectomy (N* =*)*	*Control (N* =*)*
Age (years)		
Age at diagnosis (years)		
Gender, female, *n (% n/N)*		
Disease duration (years)		
Smoking status, *n (% n/N)*		
Current		
Former		
BMI (kg/m^2^)		
ASA physical status classification grade > II, *n (% n/N)*		
PSC, *n (% n/N)*		
Family history of IBD, *n (% n/N)*		
Medication at baseline		
No medication, *n (% n/N)*		
Topical therapy, *n (% n/N)*		
5-ASA, *n (% n/N)*		
Systemic steroids, *n (% n/N)*		
Immunomodulators, *n (% n/N)*		
Extent of disease		
Proctitis, *n (% n/N)*		
Left-sided colitis, *n (% n/N)*		
Pancolitis, *n (% n/N)*		
Start of most recent exacerbation UC before randomisation (weeks)		

*Abbreviations*: *BMI* body mass index, *ASA* American Society of Anaesthesiologists, *PSC* primary sclerosing cholangitis, *IBD* inflammatory bowel disease, *5-ASA* 5-aminosalicylic acid, *UC* ulcerative colitis

## Analysis

### Outcome definitions

#### Primary outcome

The primary outcome measure is the 1-year total UC relapse rate, defined as:

- Both clinically and endoscopically with a total Mayo score ≥ 5 and endoscopic subscore of 2 or 3
- OR clinically in absence of endoscopy, based on review by an independent critical event committee (see below)

Relapse data was collected at the 3-, 6-, 9-, and 12-month follow-up forms and the end of study form. Clinically suspected relapses without endoscopic confirmation were evaluated by a critical event committee (CEC), consisting of an independent IBD surgeon and gastroenterologist blinded to the allocation group. The CEC members were the same for both the NL and the UK. The decision will be based on clinical information suggesting relapse (exacerbation of abdominal symptoms, increased bowel frequency and rectal bleeding) or FCP > 150 (> 4 weeks after surgery) or intensified medical therapy other than 5-ASA therapy.

#### Secondary outcomes

Secondary outcomes include:

1. Number of relapses per patient after 12 months
2. Time to first relapse defined as the time between randomisation in the control group or laparoscopic appendectomy in the intervention group and the first day of clinical symptoms of an endoscopically or clinically confirmed relapse
3. Disease activity measured with the total Mayo score at baseline and 12 months and the pMS assessed at 3, 6, and 9 months [10]. The total Mayo score consists of four components stool frequency, rectal bleeding, endoscopic appearance, and physician’s global assessment (Table 2). These items are rated from 0 to 3, resulting in a total Mayo score ranging from 0 to 12 and a pMS without endoscopic assessment ranging from 0 to 9. In the Mayo score, clinical remission is defined as a total Mayo score of 2 points or lower, with no individual subscore exceeding 1 point. Mucosal healing is defined as an absolute subscore for endoscopy of 0 or 1
4. Number of colectomies at the 1-year follow-up
5. Medication usage (no medication, topical therapy, 5-ASA, systemic steroids, immunomodulators, biologicals, small molecules, trial medication) at baseline, 3, 6, 9, and 12 months
6. HRQL measured by the EQ-5D health status questionnaire [12], the European Organisation for Research and Treatment of Cancer (EORTC) QLQ-C30 [11], and the Inflammatory Bowel Disease Questionnaire (IBDQ), at baseline, 3, 6, 9, and 12 months [13, 16]. The EQ-5D is a generic standardised measure of HRQL at the day of completion consisting of the EQ-5D descriptive system and the EuroQol visual analogue scale (EQ-VAS). The EQ-5D comprises 5 problem areas (mobility, self-care, daily activities, pain/discomfort, mood) with patients indicating whether they experience no, some, or extreme problems. The EQ-VAS is a vertical scale grading overall health status, ranging from 0 (worst imaginable health state) to 100 (best imaginable health state). Global quality of life (QoL) is assessed using two items of the global QoL dimension (items 29 and 30 in version 3.0) of the EORTC-QLQ-C30 that reflect overall health and QoL on the day of completion. These two items are 7-point response scales, ranging from 1 (very poor) to 7 (excellent). The average of these two items is estimated, which is the raw score (RS). The global QoL is scored by transforming the RS to a standardised 0–100 final scale score. If one or both items are missing, the global QoL is scored as missing. The IBDQ is a disease-specific questionnaire measuring QoL in 4 domains (bowel symptoms, systemic symptoms, social function and emotional function) over 2 weeks preceding completion. The IBDQ consists of 32 questions rated on a scale of 1–7, resulting in a total score ranging from 32 to 224. The score per domain is also estimated. If one or more items are missing, a domain and the total IBDQ are scored as missing. After inclusion of 79 patients, the protocol was amended to include a ‘global change question’ after 12 months: ‘Since the start of the study, have your UC symptoms improved overall?’

**Table 2:** Components of the Mayo score

Stool frequency	0 = Normal no. of stools for this patient 1 = 1 to 2 stools per day more than normal 2 = 3 to 4 stools per day more than normal 3 = ≥ 5 stools per day more than normal
Rectal bleeding	0 = No blood seen 1 = Streaks of blood with stool less than half the time 2 = Obvious blood with stool most of the time 3 = Blood alone passes
Mucosal appearance at endoscopy^a^	0 = Normal or inactive disease 1 = Mild disease (erythema, decreased vascular pattern, mild friability) 2 = Moderate disease (marked erythema, absent vascular pattern, friability, erosions) 3 = Severe disease (spontaneous bleeding, ulceration)
Physician rating of disease activity	0 = Normal 1 = Mild disease 2 = Moderate disease 3 = Severe disease

^a^Not included in the partial Mayo score

#### Handling missing items

If one or more items are missing to determine the outcome score (e.g. stool frequency to determine the partial and total Mayo score), the outcome (e.g. pMS) is scored as missing.

### Analysis methods

#### Primary outcome analysis

The 1-year UC relapse rate will be compared between the intervention and control groups with chi-square testing (Table 3).

**Table 3:** Primary outcome results

	Appendectomy *N* =	Control group *N* =	*P* value^1^	*Adjusted P value*^*2*^
Total relapse rate, *n* (% *n*/*N*)				

^1^Chi-square test

^2^Logistic regression

#### Additional analysis primary outcome

##### Stratified analysis, covariate adjustment, subgroup analysis

Logistic regression on the 1-year UC relapse rate will be used to (i) explore the interaction between treatment and disease location as stratification factor during randomisation and (ii) adjust for the following covariates: age at time of randomisation, gender, smoking status, extent of disease, and time between start of most recent exacerbation of UC and randomisation [17]. In addition, the interaction between treatment and country (UK vs. NL) will be exploratively addressed (Table 4).

**Table 4:** Subgroup analysis for primary outcome

	Appendectomy *N* =	Control group *N* =	*P* value for interaction
NL total relapse rate, *n* (% *n*/*N*)			
UK total relapse rate, *n* (% *n*/*N*)			

*Abbreviations*: *NL* the Netherlands, *UK* United Kingdom

##### Pragmatic ITT analysis

As described in the published study protocol, T0 lies at different time points in both groups (i.e. intervention group: T0 date of appendectomy; control group: T0 date of randomisation). To provide a pragmatic worst-case scenario for daily clinical practice, we will perform an additional analysis in which relapses occurring between dates of randomisation and appendectomy will be included as well. In this ‘pragmatic’ ITT analysis, T0 will be the randomisation date in both groups. Consequently, the follow-up time in the intervention group will be longer compared to the control group (i.e. time between randomisation date and appendectomy plus 1 year follow-up versus 1 year follow-up only).

#### Secondary outcomes analysis

The number of relapses per patient will be compared between groups with Poisson regression (Table 5), time to first relapse with Kaplan–Meier survival analysis including log-rank testing, and number of colectomies with chi-square testing (Table 5). If covariate adjustment substantially affected the primary outcome contrast, covariate adjustment will also be applied for these secondary outcomes with Poisson regression, Cox-regression, and logistic regression, respectively. If the assumption of proportional hazards seems invalid given the data, the time to first relapse will be analysed in distinct strata. Use of medication over time and by group will be descriptively reported by number and percentages (Table 6). General estimation equation will be utilised to examine the impact of intervention on medication use over time within treatment, time and the interaction between treatment, and time as model parameters.

**Table 5:** Secondary outcome results

	Baseline		3 months		6 months		9 months		12 months	
	A *N* =	C *N* =	A *N* =	C *N* =	A *N* =	C *N* =	A *N* =	C *N* =	A *N* =	C *N* =
Number of relapses per patient, median (IQR)										
Time to first relapse, median, (IQR)										
HRQL, median (IQR)										
EQ-5D score										
Global QoL score										
Total IBDQ score										
IBDQ: bowel symptoms										
IBDQ: systemic symptoms										
IBDQ: social function										
IBDQ: emotional function										
Mayo score, median (IQR)										
Total Mayo score										
Partial Mayo score										
Number of colectomies at one year *n* (% *n*/*N*)										

*Abbreviations*: *A* appendectomy, *C* control, *IQR* interquartile range, *HRQL* health-related quality of life, *QoL* quality of life, *IBDQ* Inflammatory Bowel Disease Questionnaire

Results will be marked with one asterisk (*) if *P *< 0.05

**Table 6:** Medication usage (general estimation equation)

	Baseline		3 months		6 months		9 months		12 months	
	A *N* =	C *N* =	A *N* =	C *N* =	A *N* =	C *N* =	A *N* =	C *N* =	A *N* =	C *N* =
No medication, *n* (% *n*/*N*)										
Topical therapy, *n* (% *n*/*N*)										
5-ASA, *n* (% *n*/*N*)										
Systemic steroids, *n* (% *n*/*N*)										
Immunomodulators, *n* (% *n*/*N*)										
Biologicals, *n* (% *n*/*N*)										
Trial medication, *n* (% *n*/*N*)										

*Abbreviations*: *A* appendectomy, *C* control, *5-ASA* 5-aminosalicylic acid

Additional generalised linear mixed models will be applied to investigate whether a different pattern of change over time exists between the two study arms in the Mayo score and the IBDQ, EQ-5D, EQ-VAS, and EORTC QLQ-C30 [18]. Best fitting covariance structures among repeated data will be based on visual inspection and Akaike’s information criterion. Baseline scores will be included as covariates in the models of repeated data.

To assess the clinical relevance of changes in the IBDQ, a clinical minimally important difference in IBDQ will be determined using a clinical anchor-based method. The minimally important difference will be calculated from the difference in IBDQ change scores of the patients answering ‘yes’ and ‘no’ to the ‘global change question’. Furthermore, the correlation coefficient between the IBDQ score and the global change question will be calculated by Pearson’s correlation method; a minimum correlation of at least 0.30 will be regarded as acceptable.

The critical *P* value of 0.05 will not be adjusted for multiple testing and all analyses of secondary outcomes should be considered exploratory. Additional analyses not mentioned in this analysis plan but performed in response to journal reviewers will explicitly be qualified as post hoc.

### Missing data

Missing data on outcome data will not be imputed. Based on the sample size calculation, a total of 164 evaluable patients (82 per study arm) are needed. Patients are evaluable if they were not excluded due to protocol violation in eligibility or consent and if the primary outcome is available. To reach the appropriate sample size and target power in the study, patients not fulfilling these evaluability criteria were replaced. Generalised linear mixed modelling of repeated data allows for missing data. Patients without any follow-up data for an outcome will not be included in the analysis of that outcome, with the reasons for this missingness counted by group and overall.

### Harms

The number and percentage of participants experiencing any adverse events (AEs) or serious adverse events (SAEs) will be presented by treatment group, and safety AT analysis will be performed (Table 7). AEs and SAEs between randomisation/surgery and 3-month follow-up will be registered. SAEs will be followed up at least until the final consequences have become clear, even if it implies that the follow-up continues beyond the planned follow-up period. For patients undergoing appendectomy, in-hospital stay (*N* nights), postoperative complications, and reinterventions will be reported. Complications of laparoscopic appendectomy will be classified according the Clavien-Dindo classification [19].

**Table 7:** Safety (reported as-treated)

	Arm A *N* =	Arm B *N* =	*P* value^1^
Total SAE, *n* (% *n*/*N*)			
Total AE, *n* (% *n*/*N*)			

*Abbreviations*: *A* appendectomy plus maintenance therapy, *B* maintenance therapy, *SAE* serious adverse event, *AE* adverse event

^1^Chi-square test

### Statistical software

Analyses will be carried out using the latest version of SPSS statistics (IBM Corp.) at the time of analysis.

## Manuscript and authorship

The steering committee of the ACCURE trial will share the results irrespective of the outcomes. The manuscript will be submitted on behalf of the ACCURE study group in alphabetical order. The coordinating investigator and principal investigator will be first and senior authors, respectively. The steering committee, other local principal investigators, physician assistants, and research nurses who were responsible for significant patient recruitment and data collection will be listed in the ACCURE study group.

## Discussion

The ACCURE trial is an investigator-initiated two-arm, multicentre, non-blinded, randomised controlled superiority trial in UC patients in complete clinical and endoscopic remission with the aim to assess whether the efficacy of laparoscopic appendectomy in addition to standard medical treatment is beneficial in maintaining remission in UC patients.

### Challenges

In the design of the trial, we faced several challenges mostly regarding accrual of the trial, which was slower than anticipated. First, accrual might have been challenging due to the narrow eligibility criteria of the trial; originally, only patients in remission treated with 5-ASA were eligible. To improve inclusion rates, the criteria were amended in 2018, by also including patients who were in remission on immunomodulators and patients who were previously treated with biologicals (> 3 months prior to inclusion). Second, when including patients in remission, the motivation for patients to participate in a trial is probably lower compared to patients with active UC. Furthermore, in daily practice, surgeons and gastroenterologists might also be less encouraged to counsel/include patients without active disease in a trial. Third, when comparing a surgical intervention with standard therapy in a randomised controlled setting, the majority of patients participating in the trial might opt for an appendectomy because they are already receiving the standard treatment. Randomised controlled trials are still seen as the gold standard. However, to increase accrual and prevent selection bias, a patient preference model might have been more suitable when comparing a surgical intervention versus medical therapy. Fourth, during the COVID pandemic the trial was paused for almost a year.

Another problem was that not all patients underwent endoscopy after 1 year of follow-up. According to the published protocol, the primary outcome is the 1-year UC relapse rate, defined both clinically and endoscopically as a Mayo-score ≥ 5 with an endoscopy score of 2 or 3. This issue was especially pronounced in patients without symptoms, making it difficult to persuade them to undergo colonoscopy. However, for patients presenting symptoms of a flare, it was not always possible to perform a colonoscopy. In the meeting on November 20, 2018, the DSMC advised to install a CEC to evaluate clinically suspected relapses without endoscopic confirmation. The advice was submitted to the Medical Ethics Review Committee for permission and granted on November 13, 2019. In addition to endoscopically proven relapses, the CEC also evaluated all clinically suspected relapses based on clinical information. To qualify as relapse, an exacerbation of symptoms and rectal bleeding or FCP > 150 (> 4 weeks after surgery) had to be observed, or medical therapy other than 5-ASA therapy had to be intensified. Finally, as the trial ran for a long period of time, daily clinical practice might have changed during the years. However, most developments were in the field of biologics, and these patients were not eligible for this trial.

### Future perspectives

This update contains the predefined SAP for the ACCURE trial. By publishing the SAP, we aim to increase the transparency of data analyses. The outcomes of this study will provide insight into the role of appendectomy in the clinical course of UC. For this study, an IBD team was identified in every participating hospital, which could lead to improved communication and collaboration between different hospitals in future research. This will facilitate future research projects, and we have learned during this project that close collaborations are indispensable to carry out large projects aiming to improve the treatment of UC.

### Trial status

Recruitment and randomisation concluded in September 2022. The final follow-up of participants is scheduled for completion in November 2023.

### Supplementary Information

**Supplementary Material 1.**

## Data Availability

Details regarding protocol amendments for the ACCURE trial can be provided upon request.

## Abbreviations

5-ASA: 5-Aminosalicylic acids
AE: Adverse event
ASA: American Society of Anaesthesiologists
AT: As-treated
BMI: Body mass index
CEC: Critical event committee
CONSORT: Consolidated Standards of Reporting Trials
CRC: Colorectal cancer
DSMC: Data Monitoring and Safety Committee
ECCO: European Crohn’s and Colitis Organisation
EORTC: European Organisation for Research and Treatment of Cancer
EQ-VAS: EuroQol visual analogue scale
FCP: Faecal calprotectin
HGD: High-grade dysplasia
HRQL: Health-related quality of life
IBD: Inflammatory bowel disease
IBDQ: Inflammatory Bowel Disease Questionnaire
IQR: Interquartile range
ITT: Intention-to-treat
NL: The Netherlands
pMS: Partial Mayo score
PSC: Primary sclerosing cholangitis
QoL: Quality of life
RS: Raw score
SAE: Serious adverse event
SAP: Statistical analysis plan
UC: Ulcerative colitis
UK: United Kingdom
